# Tandem Lewis Pair Polymerization and Organocatalytic Ring-Opening Polymerization for Synthesizing Block and Brush Copolymers

**DOI:** 10.3390/molecules23020468

**Published:** 2018-02-21

**Authors:** Xing-Yu Sun, Wei-Min Ren, Si-Jie Liu, Yin-Bao Jia, Yi-Ming Wang, Xiao-Bing Lu

**Affiliations:** State Key Laboratory of Fine Chemicals, Dalian University of Technology, 2 Linggong Road, Dalian, 116024, China; dut_xysun@163.com (X.-Y.S.); lsj21207108@163.com (S.-J.L.); jybks1982@126.com (Y.-B.J.); chemwym@dlut.edu.cn (Y.-M.W.)

**Keywords:** Lewis pair polymerization, click reaction, organocatalytic ring-opening polymerization, block copolymers, brush copolymers

## Abstract

Lewis pair polymerization is a powerful method for preparing soluble polymers bearing pendant active vinyl groups by directly polymerizing dissymmetric divinyl polar monomers. Herein, we present a strategy for synthesizing block and brush copolymers via tandem Lewis pair polymerization of methacrylates, “thiol-ene” click reaction and organocatalytic ring-opening polymerization of lactide.

## 1. Introduction

Since the concept “frustrated Lewis pairs” (FLPs), consisted of sterically encumbered Lewis acid and Lewis base pairs, was first described by Stephan and Erker [1], the application of FLP in activating various inert molecules such as CO_2_ has received intensive attention in recent years [2,3,4,5,6,7]. The first attempt of using the alane-based classical or frustrated Lewis pairs for polymerizing various polar monomers appeared in 2010 by Chen′s group [8,9]. In these systems, the structure of Lewis base had a drastic effect on the catalytic activity and polymerization behaviors [10,11,12,13]. In 2013, Amgoune and co-workers described the application of Zn(C_6_F_5_)_2_-based Lewis pairs in ring-opening polymerization of lactide and ε-caprolactone, affording well-defined high molecular weight cyclic polyesters or cyclic block copolymers [14]. Independently, Dagorne et al. performed the ring-opening polymerization of β-butyrolactone, lactide, and trimethylene carbonate mediated by neutral and cationic N-heterocyclic carbene zinc adducts and the BnOH/Zn(C_6_F_5_)_2_ binary mixture [15]. Subsequently, our group reported that the frustrated Lewis pairs consisted of N-heterocyclic olefins (NHOs) and Al(C_6_F_5_)_3_ were highly active in polymerizing various polar monomers such as methyl methacrylate (MMA), *^n^*butyl methacrylate (BMA), *N*,*N*-dimethylacrylamide (DMAA) and *N*,*N*-diphenylacrylamide (DPAA) at ambient temperature, affording high molecular weight polymers with relatively narrow distributions [16]. Notably, these frustrated Lewis pairs were discovered to be very efficient in polymerizing dissymmetric divinyl polar monomers at the methylacrylic C=C bond in completely regioselective manner at mild conditions, affording soluble polymers bearing pendant active vinyl groups with high molecular weight and narrow polydispersity (*M*_w_*/M*_n_ < 1.5) [17]. 

Unfortunately, the Lewis pair polymerization could not synthesize block copolymers by stepwise addition of two different monomers. ESI-TOF MS study confirmed that the resultant polymer included NHO as the initiation group bound to one end of a polymer chain and an unexpected six-membered ring lactone chain-end, which was formed from the nucleophilic backbiting of the polymeric anion to the carboxyl carbon of the adjacent unit, in companion with the release of the methoxyl group [16]. Recently, Rieger and coworkers presented the catalytic polymerization of diverse Michael-type monomers with high precision by using simple but highly active combinations of phosphorus-containing Lewis bases and organoaluminum compounds [18]. The interacting Lewis pairs afforded the living polymerization of a broad variety of sterically demanding and functionalized monomers with high initiator efficiencies up to 95%.

In the present contribution, we report the synthesis of block and brush copolymers via tandem Lewis pair polymerization and organocatalytic ring-opening polymerization.

## 2. Results and Discussion

### 2.1. Synthesis of PMMA-b-PLA.

**Scheme 1:**
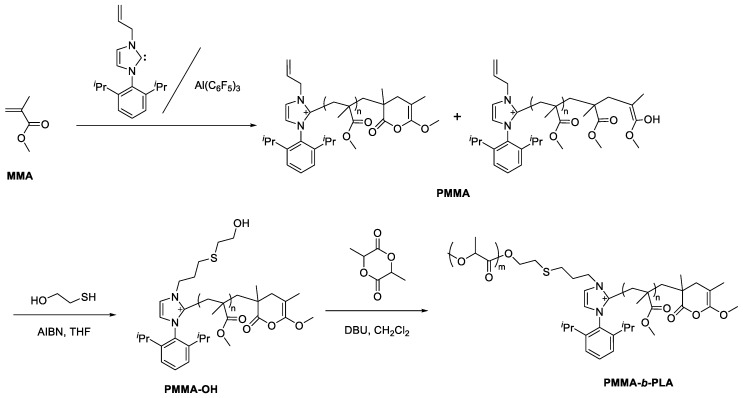
Synthesis of PMMA-*b*-PLA via tandem Lewis pair polymerization and organocatalytic ring-opening polymerization.

**Figure 1:**
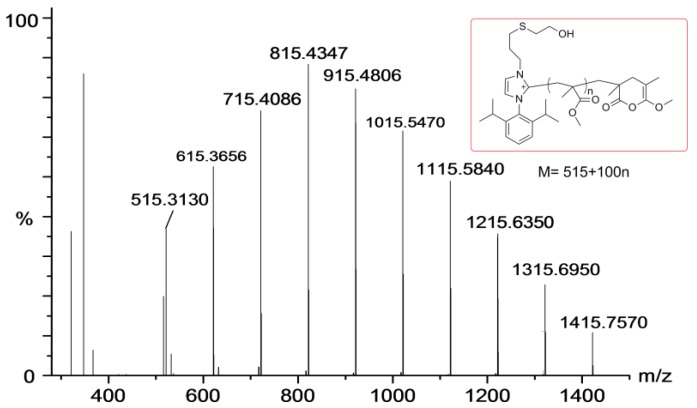
ESI-TOF MS spectrum of PMMA with hydroxyl group at the chain end.

**Figure 2:**
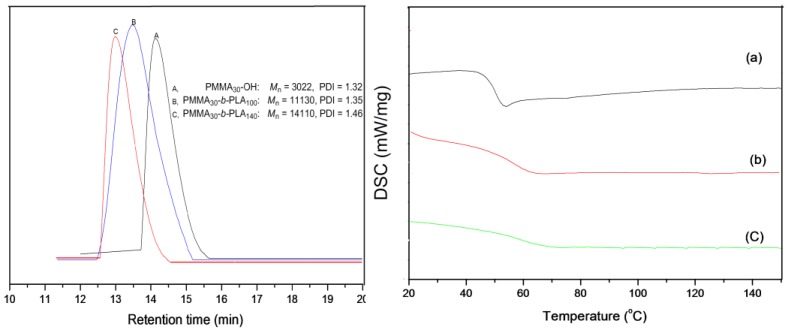
GPC spectra (left) of PMMA-*b*-PLA copolymers, and DSC thermograms (right) of PMMA-*b*-PLA copolymers with a certain amount of PMMA segment (*M*_n_(PMMA) = 3022) and different PLA segments: (a) *M*_n_(PLA)= 11088; (b) *M*_n_(PLA) = 8108; (c) *M*_n_(PLA) = 1959.

In previous paper [11], we have demonstrated that the production of lactone end resulted in complete deactivation in polymer chain propagation for Al(C_6_F_5_)_3_/Lewis base mediated polymerization of MMA. The Lewis pair consisting Al(C_6_F_5_)_3_ and N-heterocyclic carbene (NHC, compound 3) with a C=C bond group mediated polymerization of MMA predominately afforded PMMA with NHC bounded to one end of a polymer chain and a six-membered lactone ring appeared at another chain end. The vinyl group in NHC end of the resultant PMMA was transferred to hydroxyl group by the click reaction with HOCH_2_CH_2_SH. The thiol-ene click reaction was clearly confirmed by ESI-TOF MS analysis, in which the species (515 + 100n) were observed (Figure 1). The resultant PMMA-OH in Scheme 1 could be directly used as macroinitiators to initiate ring-opening polymerization of lactide with DBU as catalyst at ambient temperature to produce the PMMA-*b*-PLA diblock copolymers, confirmed by ESI-TOF MS analysis (Supporting information, Figure S1). The gel permeation chromatography (GPC) analysis of the resulting copolymers with different lactide loadings demonstrated the formation of the PMMA-*b*-PLA diblock copolymers (Figure 2, left). The thermal properties of the resulting block copolymers were determined by differential scanning calorimetry (DSC) (Figure 2, right). Only a single glass-transition peak (*T*_g_) was observed for the block copolymers. There was a gradual decrease in the *T*_g_ with the length of PLA segment from racemic lactide, compared with PMMA. Table S1: PMMA_30_-OH initiatied ring openging of lactide to prepare of PMMA-*b*-PLA copolymer. 

### 2.2. Synthesis of Brush Copolymers with PMMA Backbone and Grafting PLA Chains

Lewis pair polymerization is a powerful method for polymerizing dissymmetric divinyl polar monomers in excellent regioselectivity and high reactivity at mild conditions, affording soluble polymers bearing pendant active vinyl groups with high molecular weight and narrow polydispersity [12]. The pendant vinyl groups in polymers are easily functionalized by “thiol−ene” reaction, and the resulting hydroxyl groups can be used as initiators in various ring-opening polymerization of lactones. 

**Figure 3:**
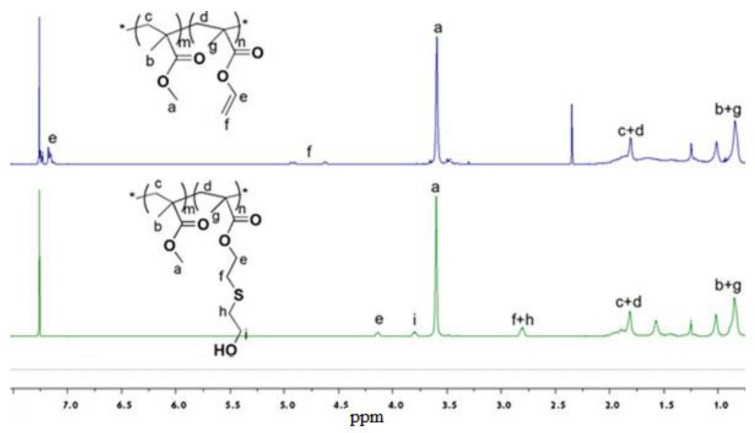
^1^H-NMR spectra of P(MMA-*co*-VMA) and P(MMA-*co*-VMA)-OH.

**Scheme 2:**
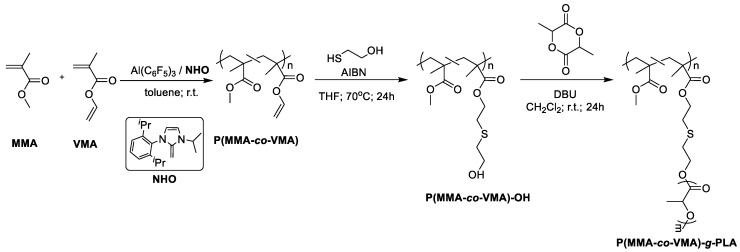
Synthesis of P(MMA-*co*-VMA)-*g*-PLA bush copolymers.

**Figure 4:**
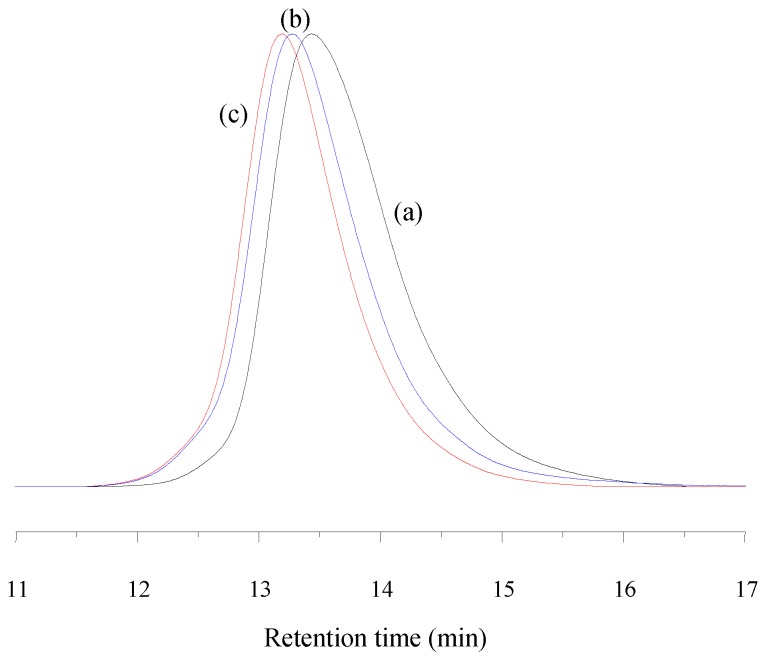
GPC spectrum of (a) P(MMA-*co*-VMA)-OH (*M*_n_ = 9200, PDI = 1.26), (b) P(MMA-*co*-VMA)-*g*-PLA_10_ (*M*_n_ = 11900, PDI = 1.29), (c) P(MMA-*co*-VMA)-*g*-PLA_20_ (*M*_n_ = 13300, PDI = 1.28).

In order to obtain well-distributed graft chains in PMMA backbone, the random copolymer P(MMA-*co*-VMA) from Al(C_6_F_5_)_3_/NHO mediated copolymerization of MMA and VMA (10:1, molar ratio) was prepared for synthesizing macroinitiators P(MMA-*co*-VMA)-OH with multi-hydroxyl sites through the reaction of P(MMA-*co*-VMA) and excessive β-mercaptoethanol in the presence of α,α-Azobis(isobutyronitrile) (AIBN) at 70 °C for 24 hours. Since MMA and VMA have the similar reactivities in this polymerization process, the monomers incorporated into the resulting copolymer exhibit a random distribution. After the click reaction of P(MMA-*co*-VMA) with β-mercaptoethanol, ^1^H-NMR spectra (Figure 3) showed that the peaks at 7.18, 4.94, and 4.63 ppm belong to the pendant C=C bonds disappeared and new peaks at 2.73, and 3.72 ppm (assigned to mercaptoethanol) appeared, indicating all the pendant double bonds have been reacted. The ring-opening polymerization of lactide initiated by P(MMA-*co*-VMA)-OH was performed under a [LA]/[-OH] feeding ratio of 10 or 20, using DBU as catalyst in CH_2_Cl_2_ at 25 °C, affording brush copolymers with PMMA backbone and grafting PLA chains (Scheme 2), ascribed to P(MMA-*co*-VMA)-g-PLA_10_ and P(MMA-*co*-VMA)-g-PLA_20_, respectively. The GPC traces of the resultant brush copolymers are shown in Figure 4, where it is apparent that an increase in lactide loading led to an increase in the copolymer′s molecular weight. Of importance, the copolymers display monomodal weight distributions with very narrow PDI values (<1.30), which demonstrates successful chain extension from the side hydroxyl groups of PMMA to afford the predesigned brush copolymers. Figure S4: ^1^H-NMR spectrum of NHO, Figure S5: ^1^^3^C-NMR spectrum of NHO.

## 3. Experimental

All syntheses and manipulations of air- and moisture-sensitive materials were performed using standard Schlenk techniques under a dry nitrogen atmosphere or an argon-filled glovebox. NMR spectra were recorded on a Bruker Avance II 400 M type (^1^H-NMR, 400 MHz; ^13^C-NMR, 100 MHz) spectrometer. High resolution mass spectra (HRMS) were recorded on a Q-TOF mass spectrometry (Micromass, Wythenshawe, UK) equipped with a Z-spray ionization source. 

Methyl methacrylate (MMA) and vinyl methacrylate (VMA) were degassed and then dried with CaH_2_ overnight, followed by vacuum distillation under reduced pressures. The purified monomers were stored in brown bottles with 5 Å molecular sieves inside a glovebox freezer at −30 °C. Al(C_6_F_5_)_3_ was prepared by ligand exchange reactions between B(C_6_F_5_)_3_ and AlEt_3_. The used N-heterocyclic olefin was prepared according to the literature methods [19].

**Figure 5:**
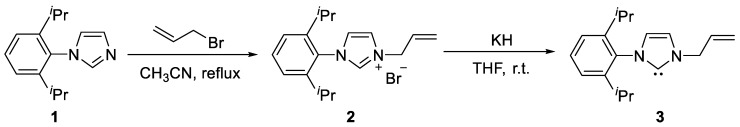
Synthesis of asymmetric carbene with a C=C bond group.

Polymer average molecular weights (*M*_n_) and molecular weight distributions (*M*_w_*/M*_n_) were determined by gel permeation chromatography (GPC) analysis using THF as the eluent with a flow rate of 1.0 mL/min, on an Agilent 1260 instrument coupled with an Agilent RI detector and equipped with two PL gel 5 μm mixed-C columns. The sample concentration was about 0.1%, and the injection volume was 50 uL. The curve was calibrated using monodisperse polystyrene standards covering the molecular weight rage from 580 to 460,000 g/mol. Low-molecular-weight polymers produced by NHO and Al(C_6_F_5_)_3_ in toluene was analyzed by electrospray ionization mass spectrometry (ESI−MS) in positive mode, using a Aglient 6224 TOF LC/MS or matrix-assisted laser. Synthesis of asymmetric carbene with a C=C bond group was shown in Figure 5.

### 3.1. Synthesis of Compound ***2***

A round-bottom flask equipped with a three-way stopcock containing 1-(2,6-bis isopropylphenyl)imidazole of 0.82 g (3.59 mmol) dissolved in 20 mL acetonitrile was purged with dry nitrogen, and 3-bromopropylene (3.95 mmol) was added into the mixture solution. The resulting mixture was refluxed over the night, and the solvent was removed under reduced pressure. The residual was purged with 10 mL diethyl ether to afford white solid. The solid was purified by column chromatography on silica gel using petrol ether/ethyl acetate (50:1-10:1, gradient elution) to give the compound 2 of 0.8 g (80% yield). ^1^H-NMR (400 MHz, DMSO-*d*_6_, in *ppm*): δ = 7.46 (t, 1H, p-Ph ), 7.43 (t, 1H, m-Ph), 7.26 (t, 1H, m-Ph), 7.23 (d, 2H, CH_2_=CH), 6.93 (t, 1H, CH=CH_2_), 2.38 (m, 2H, CH(CH_3_)_2_), 2.20 (d, 2H, *J* = 4.0 Hz, CH_2_), 1.14 (d, 12H, *J* = 8.0 Hz, CH_3_), 1.12 (d, 12H, *J* = 8.0 Hz, CH_3_).HRMS (ESI, *m*/*z*) calcd. For C_18_H_25_N_2_ [M]^+^ = 269.2018, found: 269.2200.

### 3.2. Synthesis of Compound ***3***

A Schlenk flask equipped with three-way stopcocks was successively added compound **2** (347 mg, 0.1 mmol), THF (10 mL), and then KH (44 mg, 0.11 mmol) in a nitrogen atmosphere. After the mixture solution was stirred 24 hours at ambient temperature and then filtered, the resulting precipitate was washed using THF, and dried under reduced pressure to give compound 3 (70% yield). ^1^H-NMR (400 MHz, C_6_D_6_, in *ppm*): δ = 7.35 (m, 1H, p-Ph), 7.24 (d, 2H, *J* = 8.0 Hz, m-Ph), 7.22 (s, 1H, CH=CH), 6.91 (d, 1H, *J* = 4.0 Hz, CH_2_=CH), 6.85 (s, 1H, CH=CH), 6.61(d, 1H, *J* = 4.0 Hz, CH_2_=CH ), 5.20 (m, 1H, CH=CH_2_), 2.90 (m, 2H, CH(CH_3_)_2_), 2.01 (d, 2H, J = 4.0 Hz, CH_2_), 1.28 (d, 12H, *J* = 8.0 Hz, CH_3_), 1.17 (d, 12H, *J* = 8.0 Hz, CH_3_). ^13^C-NMR (100 MHz, C_6_D_6_,): δ =146.04, 123.74, 31.83, 28.50, 24.42, 23.87, 22.92, 21.29, 17.09, 14.22,13.29. Figure S2: ^1^H-NMR spectrum of compound **3**. Figure S3: ^1^^3^C-NMR spectrum of compound **3**.

### 3.3. General Polymerization Procedures

Lewis pair polymerizations were performed in 20 mL oven-dried glass reactors inside the glovebox under ambient conditions. A predetermined amount of Al(C_6_F_5_)_3_ was first dissolved in the monomer MMA/VMA mixture (10/1, molar ratio) and 5 mL of toluene, and the polymerization was started by rapid addition of a solution of N-heterocyclic olefin in 4 mL of toluene via a gastight syringe to the mixture solution under vigorous stirring. The molar ratio of Lewis acid to NHO was fixed into 2/1 for all polymerizations. After the desired time, the reaction mixture was immediately quenched by the addition of 2 mL 5% HCl-acidified methanol. The quenched mixture was precipitated into 100 mL of methanol, stirred for 1 h and filtered. The resulting polymer was further washed with methanol and dried in a vacuum oven at 50 °C overnight to a constant weight.

### 3.4. Thiol-ene Click Reaction 

A typical procedure was started with the ratio of reagents C=C groups (in the copolymer)/[β-mercaptoethanol]/[AIBN] = 1/40/0.33 (molar ratio). Thiol-ene click reaction between P(MMA-*co*-VMA) (700 mg, 0.623 mmol of C=C group ) and β-mercaptoethanol (1.95 g, 25.0 mmol) was conducted in a 50 mL Schlenk flask under nitrogen atmosphere with 10 mL THF as solvent and AIBN (34.0 mg, 0.210 mmol) as initiator. The reaction mixture was allowed to stir for 24 h at 70 °C. Then the solvent and excessive β-mercaptoethanol were removed by rotary evaporation. The crude product was dissolved into THF and precipitated in methanol. The product was redissolved and reprecipitated for three times and dried by vacuum to a constant weight.

### 3.5. Organocatalytic Ring-Opening Polymerization

In a oven-dried 25 mL Schlenk flask under nitrogen atmosphere, macroinitiator P(MMA_10_-*co*-VMA)-OH (119 mg, 0.100 mmol of hydroxyl group) and catalyst 1,8-diazabicyclo[5.4.0]undec-7-ene (DBU) (150 mg, 0.100 mmol, 1 equiv.) were dissolved in 4 mL toluene, followed the addition of racemic lactide (1.00 or 2.00 mmol, 10 or 20 equiv.). The reaction mixture was allowed to stir for 24 h at ambient conditions. After the desired time, the mixture was precipitated into 100 mL methanol, stirred for 10 min, and then filtered. The crude product was dissolved into THF and precipitated in methanol. The product was redissolved and reprecipitated for three times, and then dried by vacuum to a constant weight.

## 4. Conclusions

We have described the successful synthesis of block and brush copolymers *via* the Lewis pair mediated polymerization of conjugated polar alkenes, the “thiol−ene” reaction of the vinyl groups in polymers, and DBU-mediated ring-opening polymerization of lactide initiated by the hydroxyl groups on the polymers. The GPC and ^1^H-NMR results demonstrated that successful side-chain extension was achieved when P(MMA-*co*-VMA)-OH was used to initiated the ring-opening polymerization of lactide. Future efforts will focus on expanding the species of substrates that are building the main or side chain, and on the increasing side chains.

## Supplementary Materials

The following are available online at http://www.mdpi.com/1420-3049/23/2/468/s1, Figure S1: ESI-TOF MS spectrum of PMMA-*b*-PLA copolymer, Figure S2: ^1^H-NMR spectrum of compound **3**, Figure S3: ^1^^3^C-NMR spectrum of compound **3**, Figure S4: ^1^H-NMR spectrum of NHO, Figure S5: ^1^^3^C-NMR spectrum of NHO, Table S1: PMMA_30_-OH initiatied ring openging of lactide to prepare of PMMA-*b*-PLA copolymer.
