# Supplementary material for: Tandem Lewis Pair Polymerization and Organocatalytic Ring-Opening Polymerization for Synthesizing Block and Brush Copolymers
Source: Molecules. 2018 Feb 21;23(2):468. doi: 10.3390/molecules23020468 (PMC6017872; doi:10.3390/molecules23020468)
Supplement: Supplementary file 1 [file molecules-23-00468-s001.pdf]

Table S1. $PMMA_{30}$-OH initiatied ring openging of lactide to prepare of PMMA-b-PLA copolymer[a]

Run	Samples	Feed[b]	$M_{n\ th}$(DA)[c]	$M_{n\ GPC}$[d]	PDI	Conv.(%)	T_g
1	$PMMA_{30}$-OH	/	3347	3022	1.32	-	90
2	$PMMA_{30}$-b-PLA_{20}	10	4787	4981	1.28	100	68
3	$PMMA_{30}$-b-PLA_{100}	50	10547	11130	1.35	100	58
4	$PMMA_{30}$-b-PLA_{140}	70	13427	14110	1.46	100	50

[a] CH_2Cl_2 2 mL, 25 °C, run 2, [LA]/DBU = 10:1, run 3, [LA]/DBU = 50:1, run 4, [LA]/DBU = 70:1, [b] Feeding ratio of [LA]/[OH], [c] $M_{n\ th}$ = $([LA]/[OH])_{Feed} \times M_{n\ LA} \times$conversion(%) + $M_{n\ PMMA30\text{-}OH}$, where $M_{n\ LA}$ is the molecular weight of LA and $M_{n\ PMMA30\text{-}OH}$ is the molecular weight of $M_{n\ PMMA30\text{-}OH.}$ [d] Determined by GPC against the standard mondispersed PMMA samples.

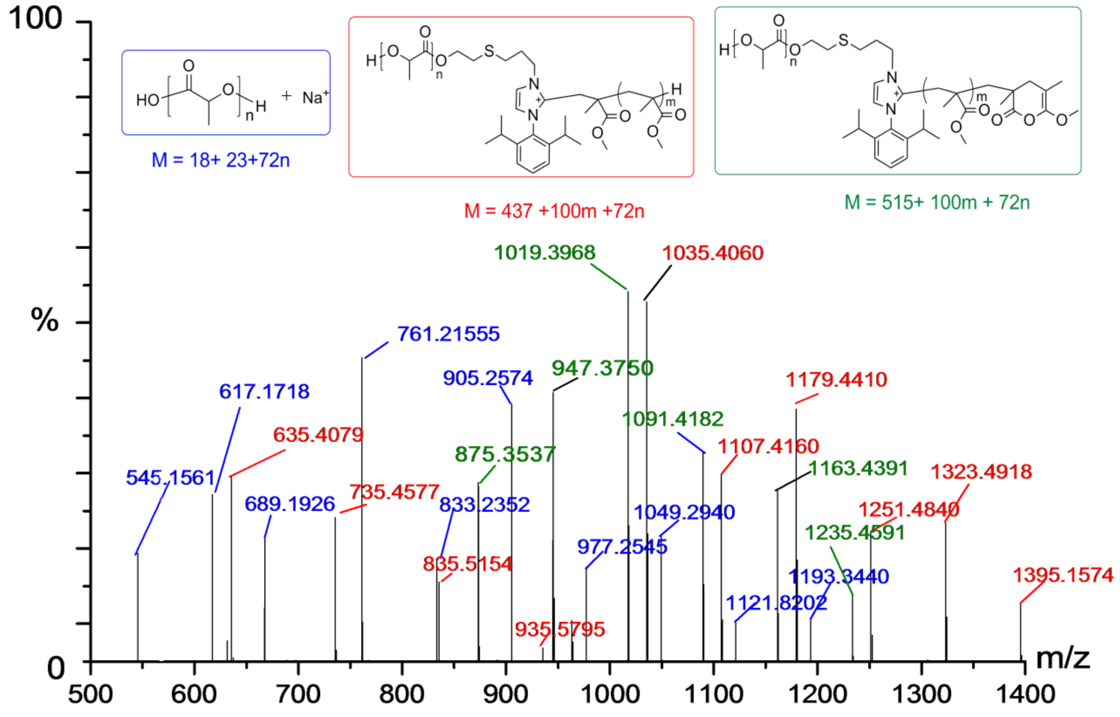

Figure S1. ESI-TOF MS spectrum of PMMA-b-PLA copolymer

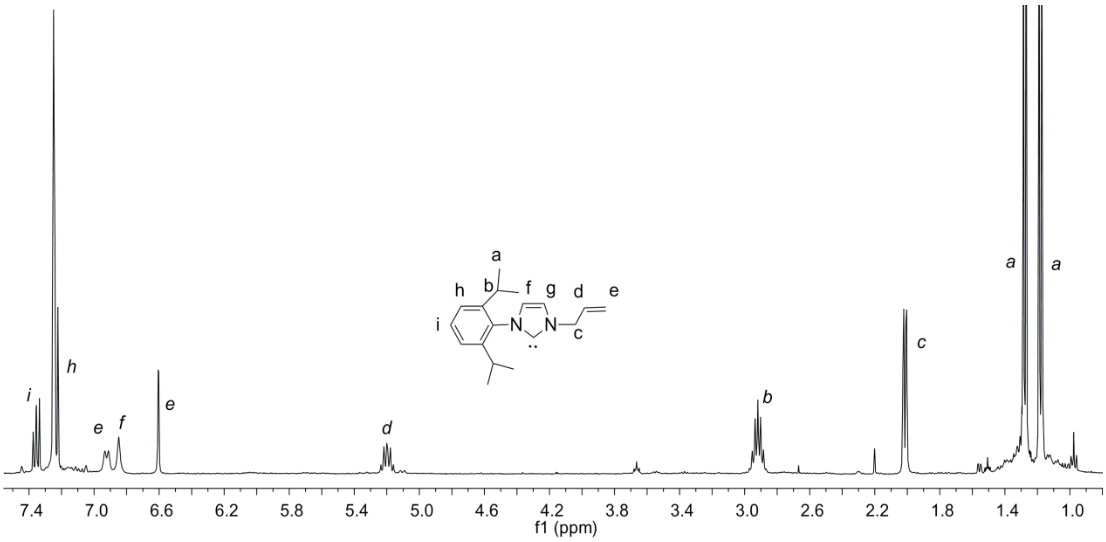

Figure S2. ^{1}H NMR spectrum of compound **3**

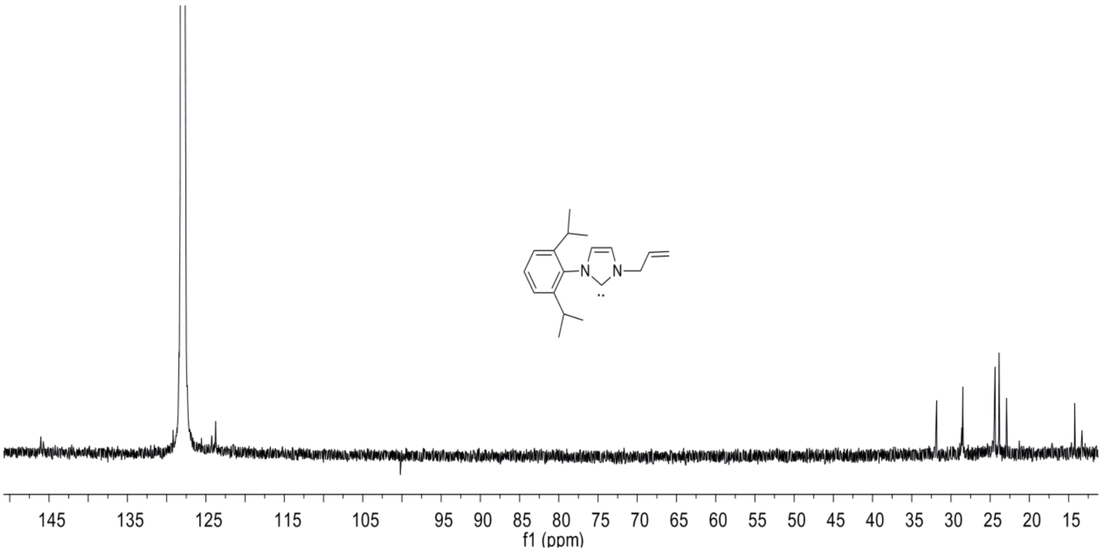

Figure S3. ^{13}C NMR spectrum of compound **3**

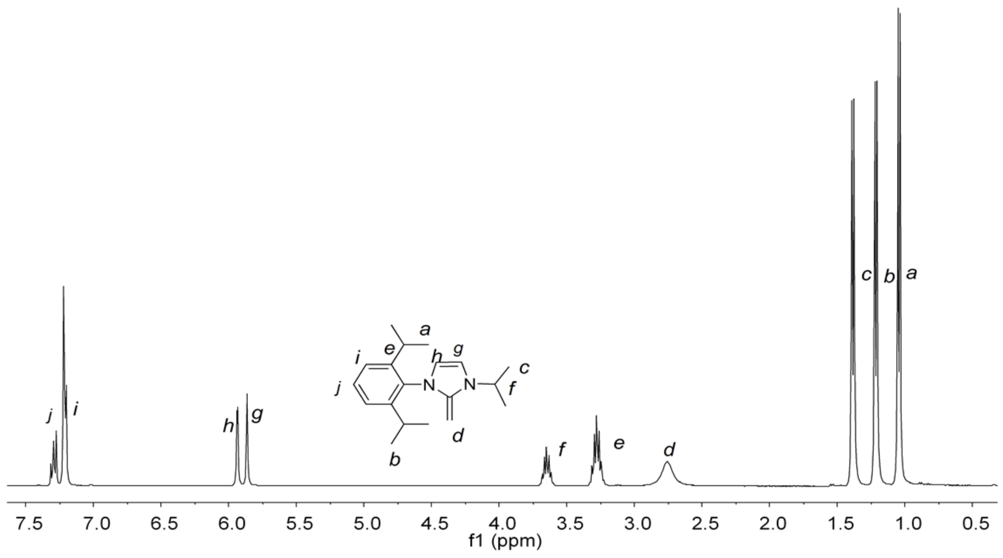

Figure S4. ^{1}H NMR spectrum of NHO

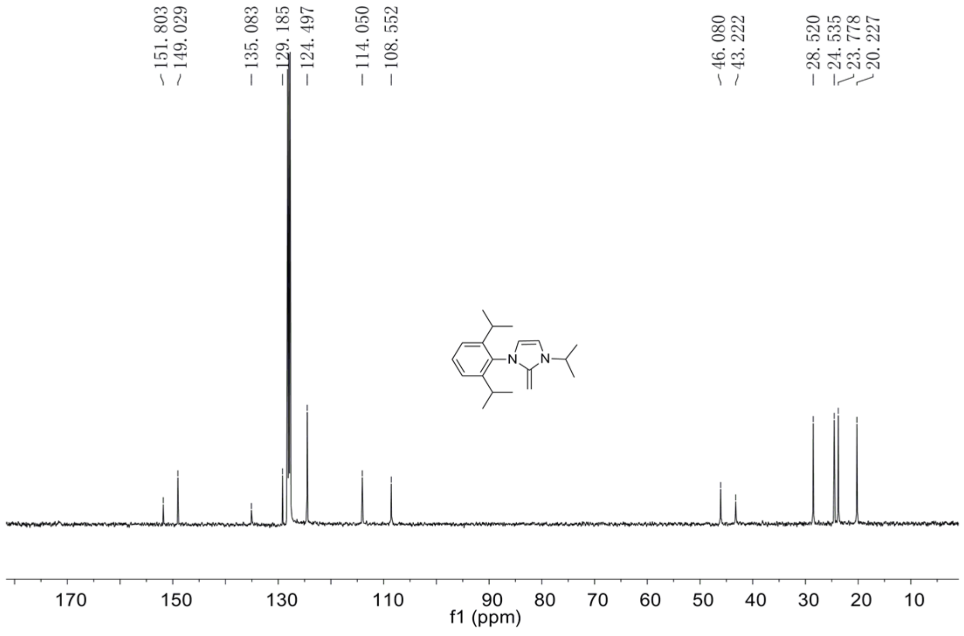

Figure S5. ^{13}C NMR spectrum of NHO
